# Rearing in a Physically Enriched Environment Affects Shoaling and Stress Responses of Zebrafish (*Danio rerio*) Exposed to Novel Conditions

**DOI:** 10.3390/vetsci12010038

**Published:** 2025-01-09

**Authors:** Valentina Gazzano, Martina Di Filippo, Rosario Licitra, Asahi Ogi, Baldassare Fronte, Maria Claudia Curadi, Angelo Gazzano

**Affiliations:** 1 Department of Veterinary Sciences, University of Pisa, 56124 Pisa, Italy; m.difilippo1@studenti.unipi.it (M.D.F.); rosario.licitra@vet.unipi.it (R.L.); maria.claudia.curadi@unipi.it (M.C.C.); angelo.gazzano@unipi.it (A.G.); 2 Istituti di Ricovero e Cura a Carattere Scientifico (IRCCS) Fondazione Stella Maris, 56128 Pisa, Italy; a.ogi@hotmail.com

**Keywords:** zebrafish, environmental enrichment, shoaling, cortisol, stress, animal welfare, behavior, refinement, lab animals, anxiety

## Abstract

The impact of enrichment on stress reduction in zebrafish (*Danio rerio*) exposed to a novel environment was assessed. Four control shoals (CTRL) and five treated shoals (TRT), each with eight fish, were observed; in TRT tanks, a PVC pipe was included (three-way tube, 11.7 × 4 cm) as enrichment for 90 days. Subsequently, fish were moved to a new tank for a shoaling test, and behavior was evaluated over periods of 0′–5′ and 5′–10′. Cortisol dissolved in water was measured before and after the test. No differences were found between the two groups in distance moved, swimming speed, or shoal acceleration. Both groups reduced interindividual distance in the second phase of the test (CTRL: t = 8.977, *p* ≤ 0.0001; TRT: t = 8.247, *p* ≤ 0.0001), though TRT fish maintained greater spacing (t = 2.292, *p* ≤ 0.05). TRT fish spent more time without contact during both phases (first: t = 2.645, *p* ≤ 0.05; second: t = 3.134, *p* ≤ 0.01), while CTRL fish reduced this time in the second phase (t = 2.991, *p* ≤ 0.05). Cortisol rose significantly in CTRL after the test (t = 2.452, *p* ≤ 0.05) but not in TRT fish. These results suggest that environmental enrichment mitigates stress, as seen by reduced cohesiveness and cortisol in TRT fish.

## Simple Summary

Laboratory animals live in environments that differ from their natural habitats, frequently experiencing high stress levels. To address this issue, environmental complexity can be increased to promote animal welfare. The aim of this research was to assess the impact of a simple environmental enrichment (a three-way orange PVC tube, 11.7 cm long and 4 cm in diameter) on stress response in fish subjected to capture and transferred into a new tank. The shoaling test evaluated some behavioral parameters associated with anxiety, and demonstrated that the enrichment had a positive effect on zebrafish stress response. These data were further confirmed by the cortisol released into the water: in fact, the subjects housed in an enriched environment did not exhibit significant increases in cortisol, unlike the control group. In summary, simple physical enrichment can help to reduce the stress response of zebrafish, contributing to improved welfare.

## 1. Introduction

The zebrafish (*Danio rerio*) has become one of the most important models in experimental biology and medicine, being used in screening studies within the fields of pharmacology, toxicology, neuroscience, physiology and genetics [1,2,3].

However, knowledge of zebrafish behavior remains limited [4,5], and laboratory housing conditions often markedly differ from natural habitats [5]. These environmental discrepancies raise concerns regarding zebrafish welfare and encourage researchers to consider ways to improve quality of life for zebrafish in captivity [5]. Optimizing welfare is essential to prevent variations in experimental results that may stem from altered physiology and behavior in response to compromised welfare [6].

Zebrafish exhibit stress and fear responses similar to mammals, making them a reliable model for studying anxiety-like behavior [7,8]. The shoaling test assesses this by measuring social behaviors like group swimming (shoaling), which is influenced by various factors affecting cohesion [9,10,11,12]. Parameters such as distance traveled, spacing between fish, time without contact, movement speed, and acceleration are used to evaluate resilience to stressors like handling and new environments [9,10,11,12,13,14]. Cortisol, released via the HPI axis in zebrafish, is a key physiological marker of stress [15,16,17]. While blood sampling for cortisol is invasive and challenging in small fish, non-invasive water cortisol measurement offers a reliable alternative, correlating with plasma and body cortisol levels [18,19].

The zebrafish is a social species that is sensitive to housing conditions [5,20,21]. Overcrowding, isolation or impoverished environments can diminish social interactions [5], and environmental manipulations can alter stress susceptibility [22]. Environmental enrichments provide sensory and cognitive stimulation [5,22,23], which promotes neuroprotection, neurogenesis and improved cognitive abilities [24].

The application of environmental enrichment to zebrafish is a recent research focus [23]. Studies indicate that zebrafish prefer enriched environments with complex structural elements over barren ones [25,26,27]. Byrd et al. [28] demonstrated that zebrafish favor pictures of pebbles and exhibit lower cortisol levels in such settings. Substrates like gravel, sand, aquatic plants, and hiding spaces are critical for stress reduction [5,6]. Enrichment also promotes exploration, reduces anxiety-like behaviors, and lowers whole-body cortisol levels, particularly under chronic unpredictable stress conditions [29].

While environmental enrichment may reduce aggressive behavior [30], it can also cause resource competition [27]. Concerns include reduced standardization of scientific data, potential impacts on water quality, and biosafety challenges, requiring strict hygiene practices [27]. Moreover, enrichment objects may hinder fish observation, complicating disease diagnosis and experimental procedures [6]. Therefore, enrichment elements must be manageable, safe, easy to sanitize [31], and carefully planned to balance animal welfare benefits with experimental requirements, avoiding interference with study outcomes [5,6].

In light of these considerations, the aim of this study was to evaluate the effectiveness of physical environmental enrichment in reducing the stress response of zebrafish employed as laboratory animals, when they are subjected to capture and placed in a new tank to carry out a shoaling test.

## 2. Materials and Methods

### 2.1. Zebrafish Management

The study was conducted on 80 wildtype (WT) adult zebrafish (AB line; 4 months old), maintained at the animal facility of the Department of Veterinary Sciences of the University of Pisa, in compliance with the European Union Directive 2010/63/EU on the protection of animals used for scientific purposes. Zebrafish have been housed in a recirculating aquaculture system (RAS) equipped with 3.5 L tanks (Tecniplast^®^ S.p.A., Buguggiate, Italy), (L. 32 cm; W. 11.2 cm; H. 16.6 cm) with an image of pebbles on the bottom. The water flow rate was set to 2 L/h, and water parameters were maintained within the following ranges: temperature, 28 ± 0.5 °C; pH 7.2–7.8; conductivity, 600–800 μS/cm; dissolved oxygen, (>5 mg/L); ammonia, (<1 mg/L); nitrites, (<0.25 mg/L); and nitrates, (>50 mg/L), according to Westerfield [32] guidelines. The room’s artificial lighting ensured a 12/12 light/dark photoperiod. Fish were fed four times daily, as per guidance from to Licitra et al. [33]. In detail, dry food (Zebrafeed 400–600 μm, Sparos^®^, Olhão, Portugal), at 7:30 and 13:00, and *Artemia salina nauplii* (AF SEP-Art^®^, INVE Aquaculture, Dendermonde, Belgium), at 10:30 and 16:00, were administered.

### 2.2. Study Design and Analysis

The experimental procedure consisted of the two following phases:

Social integration phase: the 80 fish were randomly divided into 10 groups (5 experimental, TRT, and 5 control, CTRL) and each group consisted of 8 fish (sex ratio 1:1) [6] maintained at a density of less than 3 fish/L [34]. They were maintained for 30 days [11,17,35] under the same housing conditions described in Section 2.1. Black dividers were placed between adjacent tanks to prevent visual contact, and these dividers were kept in place throughout the testing period [17]. A subject belonging to the CRTL groups died on day 25 of the integration phase, and it was not replaced by a new animal: for this reason, this tank was removed from the experimental procedure.

**Figure 1:**
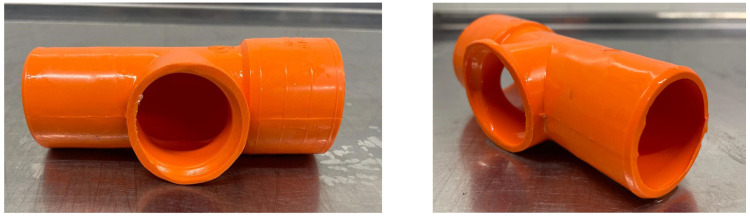
Enrichment employed: 3-way orange PVC pipe modified (Tecnomat^®^).

Enrichment introduction: the environmental enrichment introduced to TRT tanks consisted of a 3-way orange PVC pipe, 11.7 cm long and 40 mm in diameter (cod. 10035546 Tecnomat^®^; Rozzano, Italy), with three exits (two through the side branches and one from the central branch). One lateral arm was resected to facilitate the movement of the animals (Figure 1). The enrichment remained in TRT tanks for 90 days, with no change in fish management practices compared to the CTRL groups.

Cortisol sampling: On day 88 of enrichment, 80 mL of water was collected from each TRT and CTRL tank. To allow cortisol accumulation, water flow was stopped for one hour prior to sampling [28]. The same procedure was performed at the end of the shoaling test, when the animals were returned to tanks with clean water. Water samples were stored at −20 °C until processing [17]. Cortisol extraction followed the protocol described by Byrd et al. [28]: thus, water samples were processed using a C18 solid-phase extraction cartridge (500 mg, #1.02023.0001, Merck, Darmstadt, Germany), which had been preconditioned with 2 × 2 mL of absolute ethanol and then rinsed with 2 × 2 mL of distilled water. Steroids were subsequently extracted by eluting them with 2 × 2 mL of ethanol into glass tubes. The ethanol was evaporated under a fume hood, and the residue was rehydrated with 100 µL of distilled water. Diethyl ether (4 mL) was added to the suspension, which was then agitated for 20 min.

To separate the phases, the samples were centrifuged at 163× *g* for 5 min at 4 °C and then stored at −80 °C for 15–20 min to freeze the aqueous phase. The organic layer was carefully transferred into a glass vial, and the ether extraction was repeated for the remaining aqueous phase using 3 mL of diethyl ether to enhance extraction efficiency. Then, the combined ether extracts were evaporated, and the dried organic fraction containing the free steroids was dissolved in 1 mL of the buffer solution supplied with the Enzyme Immunoassay (ELISA) kit. Finally, cortisol concentrations in the extracts were measured using an ELISA kit (cod 500360; Cayman Chemical’ 1180 East Ellsworth RoadAnn, Arbor, MI 48108, USA) [28].

Shoaling Test: On day 90, the shoaling test was performed, as described by Johnson et al. [9]. The test arena was a transparent plexiglass cube (40 cm high × 40 cm wide × 40 cm long) filled with clean RAS water [17] to a total volume of 40 L and a water height of 25 cm. The temperature of the water in the tank was set to 28 °C with an aquarium heater, removed before the start of the test and reintroduced after the complete replacement of the water, before testing the next group.

Three sides of the arena were covered with white insulating tape to minimize visual disturbance from operators [9].

### 2.3. Shoaling Test Procedure

The animals were tested after their usual morning feeding, to prevent stress from food deprivation [17]. The tanks of fish to be tested were kept for two hours in the test room, to allow the animals to acclimatize. The temperature was maintained by placing a heating mat under the individual tanks, and each group was tested individually. All fish from each tank were gently removed, simultaneously, from the housing tank, using a net, and released into the arena. Behavioral assessment began once all the animals were placed in the arena. Each test session lasted 10 min, with behavioral parameters recorded in two intervals: 0–5 min and 5–10 min. This design allowed for detecting both the acute stress response to the change in environment upon transfer to the test tank, and the animals’ ability to recover from the stressful situation. To minimize additional stress, loud noises and sudden and rapid movements were avoided in the test room [17]. The sessions were recorded with a digital video camera (Basler AG, Ahrensburg, Germany), positioned above the apparatus, and the videos were analyzed with the Ethovision XT17 tracking software (Noldus Information Technology, Wageningen, Netherlands). Behavioral parameters analyzed in both test periods (0–5; 5–10 min) for all individuals of every group were:

- The distance (cm) traveled by the subjects.
- The individual distances (cm) between each subject.
- The time (s) elapsed without body contact between the subjects.
- The movement speed (cm/s) of the subjects.
- The individual acceleration (cm/s^2^) of the fish.

### 2.4. Statistical Analysis

The unit of statistical analysis was the shoal. Data were analyzed using the two-way repeated measures ANOVA and post hoc multiple comparison were evaluated using Fisher’s Least Significant Difference test. Statistical significance was set at *p* ≤ 0.05, and all analyses were conducted using GraphPad Prism 9 (Graph-Pad Software, San Diego, CA, USA).

## 3. Results

**Figure 2:**
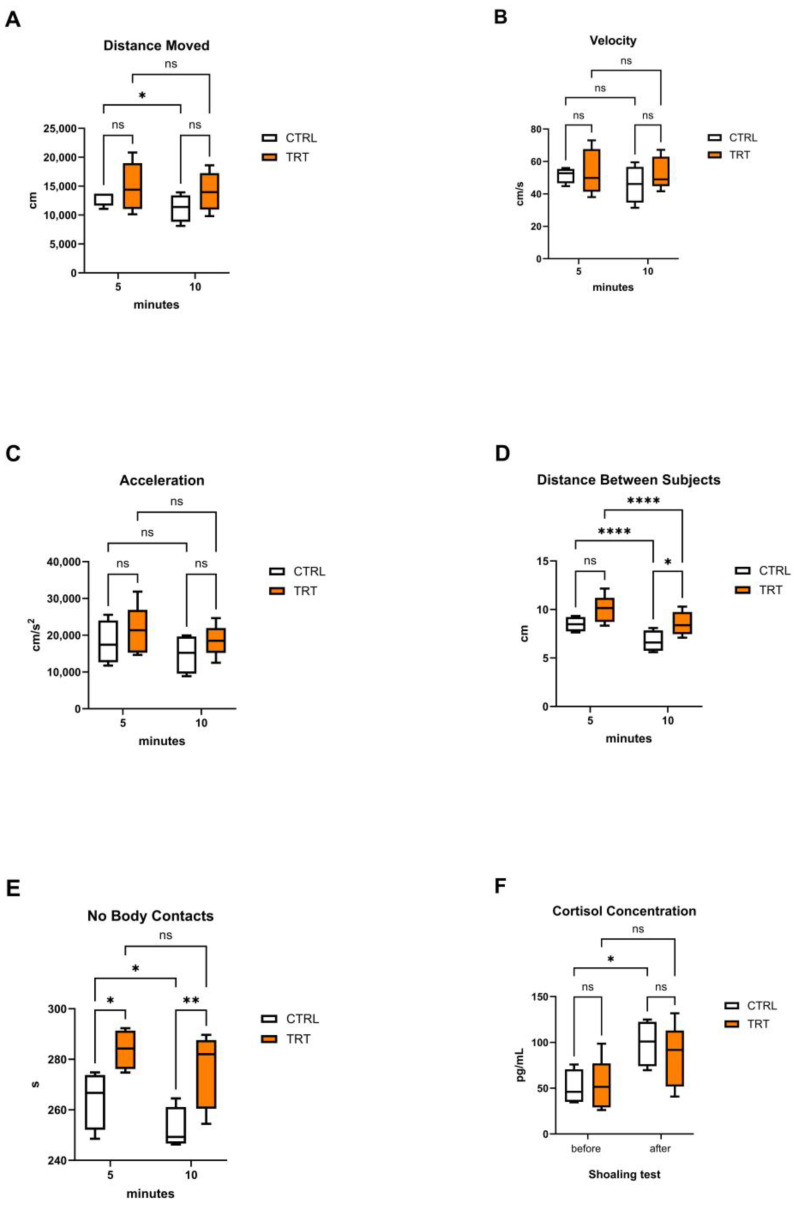
Shoaling behavior (**A**–**E**) and water cortisol concentrations (**F**) in the control (CTRL; *n* = 4 tanks of 8 fish) and treated (TRT; *n* = 5 tanks of 8 fish) groups across the two time intervals tested (from 0 to 5 and from 5 to 10 min for the shoaling test, and before and after the shoaling test for the cortisol concentration). Data are presented using box-and-whisker plots, indicating the range of the central 50% of the data, with a central line marking the median value. Lines extend from each box to capture the range of the remaining data (* *p* ≤ 0.05; ** *p* ≤ 0.01; **** *p* ≤ 0.0001).

Measurements made on each group of fish during the shoaling test are summarized in Figure 2A–E, and cortisol concentrations recorded in the tank water are given in Figure 2F (complete data sets are in the Supplementary Material Table S1: Data set).

No significant differences were observed between the CTRL and TRT groups for distance moved, swimming speed, and acceleration of the shoal (Figure 2A–C). However, within the CTRL group a significant decrease in distance moved was detected between the first (0–5 min) and second (5–10 min) phase of the test (t = 2.747; *p* ≤ 0.05) (Figure 2A).

For inter-individual distance (Figure 2D), both groups reduced their swimming distances during the second phase of the test (from 5 to 10 min) compared to the first phase (0–5 min) (CTRL: t = 8.977; *p* ≤ 0.0001; TRT: t = 8.247; *p* ≤ 0.0001). Notably, TRT fish maintained a significantly greater inter-individual distance than CTRL fish during the second phase (t = 2.292; *p* ≤ 0.05).

Regarding time spent without physical contact between individuals, the TRT groups exhibited significantly more time without contact compared to the CTRL group during the first phase (t = 2.645; *p* ≤ 0.05) (corresponding to the adaptation phase in the new tank) and the second phase of the test (t = 3.134; *p* ≤ 0.01), (Figure 2E). A statistically significant reduction in this time was found in CRTL fish between the two test periods (t = 2.991; *p* ≤ 0.05).

Finally, cortisol analysis revealed no significant increase in cortisol release in TRT groups following the behavioral test and associated handling. In contrast, CTRL fish exhibited a significant rise in cortisol levels post-test (t = 2.452; *p* ≤ 0.05).

## 4. Discussion

Stress in laboratory settings can adversely affect animals’ welfare and alter research outcomes, making it essential to implement strategies to mitigate stress. Environmental enrichment has shown promise in reducing stress in zebrafish, though it may sometimes conflict with the operational efficiency of research facilities [5,6,23].

Zebrafish are known to prefer more complex environments that offer shelter structures [27]. The aim of this research was therefore to determine if a simple and easy-to-manage form of environmental enrichment, which enhances spatial complexity, could improve the stress response in zebrafish.

Our findings indicate that enrichment reduced stress responses during handling and tank transfer for the shoaling test. Two parameters for assessing anxiety-like behavior (distance between subjects and the time without contact between subjects) showed statistically significant differences between CTRL and TRT groups. The reduction in shoal cohesion of TRT groups, observed as greater individual distance between fish in both intervals (0–5 and 5–10), suggests a lower anxiety state [9], while the entity of time without contact among TRT subjects supports the potential anxiolytic effect of the enrichment used, confirming its benefits in zebrafish welfare.

Notably, the distance between the subjects of TRT shoals was significantly greater than in the CTRL groups already in the first observation period, suggesting a positive effect of enrichment in coping with a new environmental situation. The reduction observed in the second observation period, probably due to a reduction in exploratory behavior, also remained at values significantly higher than those of the CTRL animals.

The other three behavioral parameters analyzed (the distance moved, the movement speed and the shoal acceleration), although not showing statistically significant differences between CTRL and TRT fish, exhibited a tendency to be higher in the TRT groups.

These data agree with what was found by Abozaid and Gerlai [36], who pointed out that stress and anxiety-like responses include reductions in activity (often measured as distance traveled or swim speed), elevated immobility, higher erratic movement and increased thigmotaxis. In fact, reduced swimming activity or increased time spent completely immobile allows prey to escape detection by visually hunting predators.

Similar results were obtained by Jorge and colleagues [37] in a study that used an enrichment of three PVC pipes (6.1 cm length; 3 cm external diameter; 2.5 cm internal diameter) with a dark gray color. The shoaling test produced results comparable to those in this study, although more limited, still showing an increase in the distance to the nearest neighbor in a group of animals housed in enriched tanks.

Our results were supported by cortisol concentration data. Both TRT and CTRL water cortisol increased from T0 to T1, but a statistically significant increase in cortisol levels was observed only in the CTRL, suggesting higher stress levels in this group compared to the TRT group, which appeared better able to cope with the aversive stimulus of being placed in an unfamiliar environment.

Although the data suggests environmental enrichment has a positive anti-stress effect, potential negative effects, such as increased aggression, cannot be ruled out. Literature indicates enrichment can heighten territorial aggression, especially among males [27]. Preventative strategies include maintaining balanced sex ratios in shoals to reduce aggression [38] and increasing environmental complexity [30]. Protective aggression over enrichment objects may also occur [39], necessitating careful group monitoring and removal of overly dominant individuals if needed.

Other limitations of this study are the reduced number of shoals observed, and the use of WT zebrafish. Over the years, scientific research has increasingly focused on genetically modified strains, which may exhibit behavioral and physiological differences from WT zebrafish [40]. For example, specific genetic mutations have been shown to alter social behaviors, impacting shoal structure and dynamics (e.g., mutations affecting swimming speed) [40]. Consequently, strain differences should be considered as potential biases in behavioral studies.

Further investigation, employing a greater number of shoals, could determine whether this form of enrichment also positively impacts fertilization and birth rates, factors that appear to be influenced by stress and environmental complexity [27].

## 5. Conclusions

In conclusion, this study presents the first available data on the effectiveness of a simple environmental enrichment on the welfare of zebrafish, showing that it can reduce anxiety-like behaviors and enhance adaptability to stressors, such as those involved in introductions into unfamiliar environments. In the perspective of implementing the application of the 3R principle, the use of this form of environmental enrichment can contribute to improving the coping capacity of zebrafish, reducing the variability in experimental data that can be influenced by stress.

## Data Availability

The original contributions presented in the study are included in the article; further inquiries can be directed to the corresponding author.

## Supplementary Materials

The following supporting information can be downloaded at https://www.mdpi.com/article/10.3390/vetsci12010038/s1: Table S1: Data set.
