# Supplementary material for: Rearing in a Physically Enriched Environment Affects Shoaling and Stress Responses of Zebrafish (Danio rerio) Exposed to Novel Conditions
Source: Vet Sci. 2025 Jan 9;12(1):38. doi: 10.3390/vetsci12010038 (PMC11769135; doi:10.3390/vetsci12010038)
Supplement: Supplementary file 1 [file vetsci-12-00038-s001.zip › vetsci-3322521-supplementary.pdf]

	Velocity (cm/s) 0-5 min		Distance (cm) 0-5 min	
	CTRL	TRT	CTRL	TRT
G1	44,7489	62,225175	11064,19	17152,175
G2	52,079075	38,057625	13691,1125	10129,2988
G3	53,4896125	73,0011	13708,2625	20820,0875
G4	55,99655	44,6177875	13346,8425	11973,98
G5		49,814		14378,8125

	Velocity (cm/s) 5-10 min		Distance (cm) 5-10 min	
	CTRL	TRT	CTRL	TRT
G1	31,4072625	58,7214375	8136,86625	15898,525
G2	44,2488875	41,606175	11849,2125	9818,9725
G3	47,9003625	67,1767875	10958,79	18615,8875
G4	59,530375	47,9712125	13922,4838	12071,5613
G5		48,9550625		13973,15

Average values of the shoaling test

Distance BS (cm) 0-5 min

CTRL	TRT
7,64080679	9,111401071
8,0669325	12,15466429
8,90454	10,14239857
9,33067143	8,328912143
	10,26633607

No Body Contact (s) 0-5 min

CTRL	TRT
262,786875	284,25175
274,805875	274,78125
270,6965	290,40625
248,570625	277,536125
	292,3

Distance BS (cm) 5-10 min

CTRL	TRT
5,60761	7,817846786
6,13470179	10,28638179
7,07308571	9,186116429
8,10429571	7,101881429
	8,375958214

No Body Contact (s) 0-5 min

CTRL	TRT
246,332625	282,007
264,519375	254,48375
250,9035	285,573
247,681625	266,39575
	289,691375

Acceleration (cm/s²) 0-5 min	
CTRL	TRT
11756,5163	21355,075
15383,1588	15850,19625
19420,5	21956,3375
25555,9	31833,675
	14639,3

Cortisol (pg/ml) T0	
CTRL	TRT
34,5	51,4
36,7	31,9
75,8	98,6
55,5	26,2
	55,5

Acceleration (cm/s²) 5-10 min	
CTRL	TRT
8851,7075	18467,9125
11667,5938	19264,125
19965,975	24650,6
18740,2375	17879,0475
	12519,29125

Cortisol (pg/ml) T1	
CTRL	TRT
69,7	40,9
86,9	94
115	91,8
124,9	131,8
	62,6
